# The treatment with or without prednisolone in adults aged ≥ 70 years with myasthenia gravis: a retrospective cohort study

**DOI:** 10.3389/fneur.2026.1853517

**Published:** 2026-07-29

**Authors:** Kazuo Iwasa, Yutaka Furukawa, Hiroaki Yoshikawa, Moeko Noguchi-Shinohara, Kenjiro Ono

**Affiliations:** 1 Department of Health and Medical Sciences, Ishikawa Prefectural Nursing University, Kahoku, Japan; 2 Department of Neurology, Kanazawa University Graduate School of Medical Science, Kanazawa, Japan; 3 National Hospital Organization Kanazawa Medical Center, Kanazawa, Japan; 4 Health Service Center, Kanazawa University, Kanazawa, Japan

**Keywords:** calcineurin inhibitors, myasthenia gravis, older patients, prednisolone, prognosis

## Abstract

**Introduction:**

This retrospective, single-center cohort study aimed to compare clinical outcomes, including time to minimal manifestations (MM)—defined as the absence of functional limitations from MG symptoms in daily activities, even with mild muscle weakness on examination—or better status, between patients aged ≥70 years with MG treated with PSL alone or with calcineurin inhibitors (CNIs), and those treated with CNI alone.

**Methods:**

Twelve patients with acetylcholine receptor antibody (AChR-Ab)-positive MG aged ≥70 years were included (the PSL group, *n* = 7; the CNIs group, *n* = 5). Changes in the MG Activities of Daily Living scale (MG-ADL) and Quantitative MG (QMG) scores from admission to discharge were evaluated using least-squares mean estimates. Longitudinal changes in AChR-Ab titers over one year were analyzed using a mixed-effects model for repeated measures. The time to MM was assessed using Kaplan–Meier analysis.

**Results:**

The MG-ADL scores improved in both groups, with a greater change in the PSL group. QMG scores improved in the PSL group, while no significant change was observed in the CNIs group; however, discharge QMG scores and changes did not differ significantly between the groups. No differences were observed in AChR-Ab titers or the cumulative incidence of MM. All patients in both groups achieved MM or better status within one year.

**Discussion:**

In this cohort of patients aged ≥70 years with MG, favorable outcomes were observed, regardless of PSL use. These findings highlight the clinical heterogeneity among older patients with MG and underscore the need for individualized treatment approaches.

## 1 Introduction

Myasthenia gravis (MG) is an autoimmune disorder of the neuromuscular junction caused by antibodies targeting postsynaptic membrane proteins, such as the acetylcholine receptor (AChR), muscle-specific tyrosine kinase, and low-density lipoprotein receptor-related protein 4. Clinically, MG is characterized by fatigable weakness of the ocular, facial, bulbar, respiratory, and limb muscles (1).

Recent epidemiological data indicate a demographic shift toward older onset, with individuals aged ≥ 70 years accounting for 26.8% of MG cases (2). MG is a heterogeneous disease that varies according to age at onset in terms of clinical presentation, immunological profile, thymic pathology, treatment response, and prognosis (1, 3). Traditionally, MG has been categorized as early-onset (< 50 years) or late-onset (≥ 50 years). Additionally, the term “very late-onset” is sometimes used to classify patients aged ≥65 years (1, 4–6).

Very late-onset MG is often associated with poor outcomes in the absence of immunotherapy (4) and typically requires intensive immunosuppressive treatment (5, 7). However, some studies have reported no significant differences in clinical outcomes between tacrolimus monotherapy and tacrolimus combined with corticosteroids in this population (6). Furthermore, positive treatment responses have been observed in late-onset MG, regardless of whether corticosteroids or other potent immunosuppressants are used (8–13).

Glucocorticoids, such as prednisolone (PSL), are commonly used in the treatment of MG but are associated with substantial adverse effects. These include osteoporosis, an increased risk of fracture, and other complications, particularly in older adults (14–16). Daily oral PSL doses exceeding 5 mg are associated with a higher risk of fractures within 3–6 months of initiation and may negatively affect long-term prognosis (17, 18).

Recent reports suggest that effective immunotherapy without PSL is feasible in patients aged ≥ 70 years (8, 11, 13). Avoiding PSL in selected older patients may reduce the medication burden and help minimize treatment-related adverse effects, potentially improving quality of life.

In this context, the aim of this retrospective, single-center cohort study was to descriptively compare clinical outcomes, including the time to achieve minimal manifestations (MM) or better status (MM, pharmacological remission, or complete stable remission), where MM was defined as the absence of functional limitations attributable to MG symptoms during daily activities, including household chores, hobbies, and personal care, that were performed independently before the onset of MG, despite possible mild muscle weakness on physician examination, between patients aged ≥70 years with MG treated with prednisolone (PSL) with or without calcineurin inhibitors (CNIs) and those treated with CNI alone.

## 2 Methods

### 2.1 Participants

A total of 46 consecutive patients who were newly diagnosed with acetylcholine receptor antibody (AChR-Ab)-positive myasthenia gravis (MG) and admitted to our hospital between 2008 and 2018 were screened. “Newly diagnosed” MG was defined as patients receiving their first definitive diagnosis of MG, regardless of whether initial treatment was initiated at another institution before admission to our hospital. All patients with MG provided written informed consent for the use of their clinical data during hospitalization and outpatient visits.

MG was diagnosed based on clinical fatigability, a positive AChR-Ab level (≥0.3 nM) measured by radioimmunoassay, a decremental response on repetitive nerve stimulation, and/or a positive edrophonium test.

**Figure 1:**
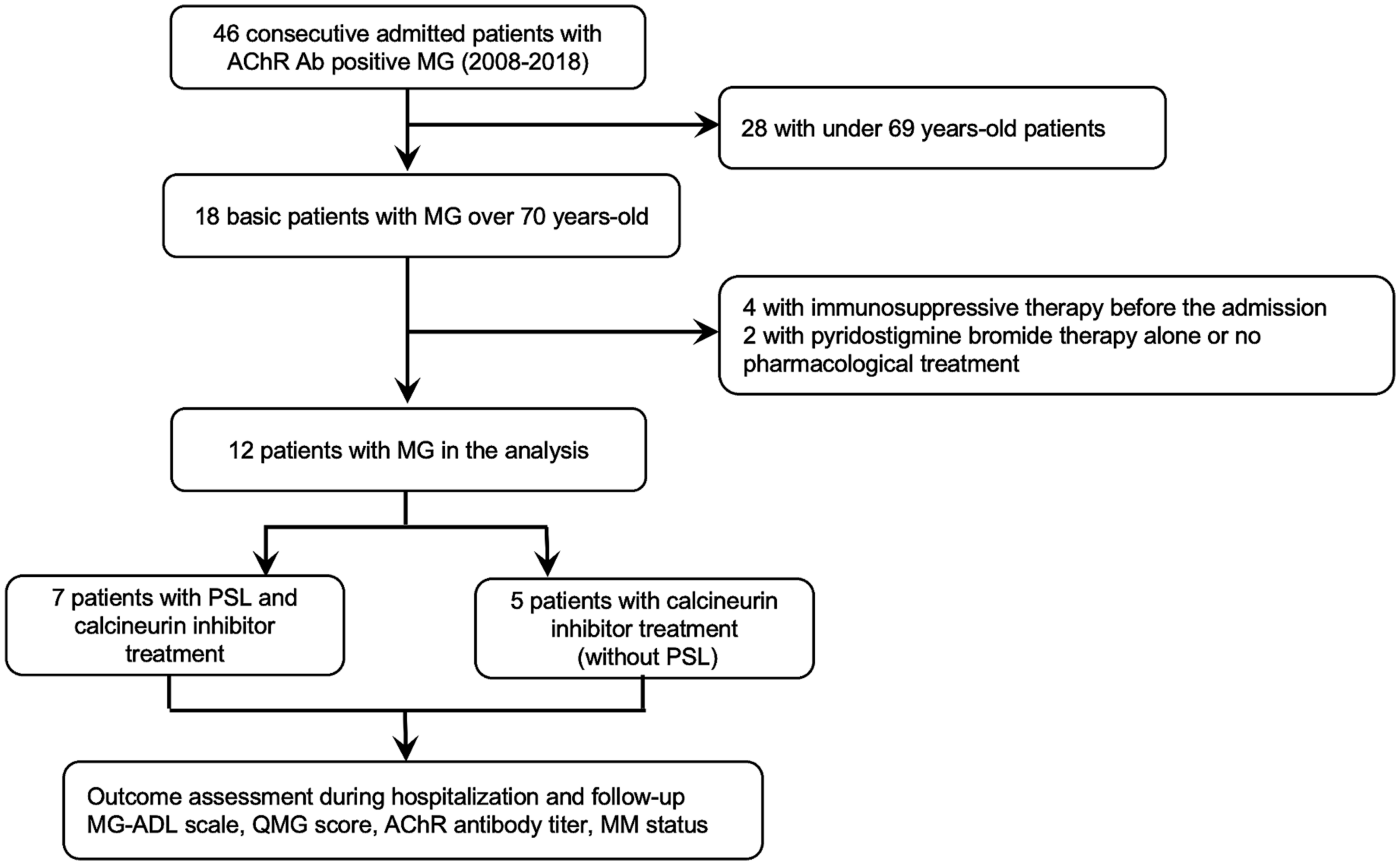
Flowchart showing the enrollment of study participants and grouping for analysis. AChR-Ab, acetylcholine receptor antibody; MG, myasthenia gravis; PSL, prednisolone; MG-ADL, myasthenia gravis activities of daily living scale; QMG, quantitative myasthenia gravis; MM, minimal manifestation.

During the study period, no patient aged 65–69 years at our institution received calcineurin inhibitor (CNI) monotherapy. In contrast, patients treated with CNI monotherapy were aged ≥74 years. Because the inclusion of patients aged 65–69 years would have introduced substantial age-related treatment bias, the analysis was restricted to patients aged ≥70 years at disease onset. Patients were excluded if they had received immunosuppressive therapy before admission (*n* = 4); PSL alone (*n* = 2), PSL plus CNI (*n* = 1), or PSL combined with CNI or plasma exchange (*n* = 1). Two additional patients were excluded because they had not received immunosuppressive therapy: one was treated with pyridostigmine bromide alone, and the other did not receive any pharmacological treatment (Figure 1). A total of 12 patients met the inclusion criteria. The median follow-up period from treatment initiation was 361.0 days (range: 79.0–374.3 days).

In Japan, immunosuppressants for MG are limited to CNIs. Eleven of the 12 patients received CNIs during follow-up, either tacrolimus (*n* = 10) or cyclosporine (*n* = 1). One patient received PSL alone because cyclosporine was discontinued due to a hemi-kidney. Seven patients who received additional PSL were assigned to the PSL group, and the remaining five were assigned to the CNIs group. PSL administration was avoided in the CNIs group because of comorbidities and age-related risks (Supplementary Table 1).

### 2.2 Monitoring of clinical course

Clinical severity was assessed at admission and discharge using the quantitative MG (QMG) and MG activities of daily living (MG-ADL) scores. Changes in scores were calculated as follows:

ΔQMG score = QMG score at admission - QMG score at discharge.

ΔMG-ADL score = MG-ADL score at admission - MG-ADL score at discharge.

Disease severity on admission was classified according to the Myasthenia Gravis Foundation of America (MGFA) classification system. Post-intervention MGFA status was used to determine the cumulative incidence of MM or better, including MM status, pharmacological remission, and complete stable remission. MM was defined as the absence of functional limitations attributable to MG symptoms during daily activities, including household chores, hobbies, and personal care, that were performed independently before the onset of MG. However, cases with mild muscle weakness detected on physician examination were also categorized as MM. In this cohort study, the endpoint was the time from treatment initiation to the first attainment of MM or better status.

### 2.3 AChR antibody titer reduction

Serum AChR-Ab titers were measured at baseline (pretreatment) and at 3, 6, and 12 months after treatment. The decremental rate (DR) of AChR-Abs was calculated as follows:


$$\begin{array}{l} \mathrm{DR}-\text{AChR}\;\mathrm{Ab}\;(\%)=[(\begin{array}{l} \text{pretreatment titer}–\\ \text{posttreatment titer} \end{array})\times 100]/\\ \text{pretreatment titer}. \end{array}$$

### 2.4 Statistical analysis

All statistical analyses were conducted using Prism version 10.1.1 for macOS (GraphPad Software, LLC, CA, USA) or R version 4.4.0 (The R Foundation for Statistical Computing, Vienna, Austria) through EZR 1.67 (Saitama Medical Center, Jichi Medical University, Japan) (19).

Demographic and clinical differences between the PSL and CNIs groups were analyzed using Fisher’s exact test and the Mann–Whitney U test.

Values for QMG and MG-ADL scores at discharge were assumed to be missing at random and were imputed using multiple imputation via chained equations with the *Amelia* package in R. Fifty imputed datasets were analyzed using generalized linear models adjusted for fast-acting treatment (FT) and baseline QMG or MG-ADL scores. Least-squares (LS) mean changes in QMG and MG-ADL scores were estimated using the *emmeans* package.

Longitudinal changes in DR-AChR Ab titers from baseline to 12 months post-treatment were analyzed using a mixed-model repeated-measures (MMRM) approach with the *mmrm* package in R. The model included group, month, and their interaction as fixed effects, pretreatment titer as a covariate, and subject as a random effect. Monthly LS mean changes and standard errors were estimated, and group differences were evaluated. Sensitivity analyses included a complete-case comparison and analysis of covariance (ANCOVA) using a generalized linear model.

The cumulative incidence of MM or better achievement within one year was analyzed using Kaplan–Meier estimates and log-rank tests. Statistical significance was set at *p* < 0.05.

## 3 Results

### 3.1 Participants’ characteristics

The demographic and clinical characteristics of the 12 patients with MG are summarized in Table 1.

**Table 1:** Demographic data of the 12 patients with myasthenia gravis aged ≥70 years (at the time of admission).

Characteristic	Patients with PSL treatment (*n* = 7)	Patients without PSL treatment (*n* = 5)	*p*-value
Sex (female: male)	2:5	5:0	0.028^b^
Age (years)	73.0 (70–78)^a^	82.0 (76.5–84.5)^a^	0.018^c^
Duration of admission (days)	65 (42.0–80.0)^a^	38 (31.5–52.5)^a^	0.115^c^
QMG score on admission	11.0 (10.0–13.0)^a^	10.0 (6.0–13.0)^a^	0.511^c^
MG-ADL score on admission	6.0 (2.0–6.0)^a^	6.0 (4.0–8.5)^a^	0.735^c^
MGFA classification on admission	I = 0 IIa:IIb:IIIb = 4:1:2	I = 1 IIa:IIb = 2:2	0.417^b^
AChR antibody level on admission (nmol/L)	24.4 (10.1–39.0)^a^	25.2 (13.6–38.9)^a^	0.876^c^

QMG, quantitative myasthenia gravis, MG-ADL, myasthenia gravis activities of daily living scale, MGFA, The Myasthenia Gravis Foundation of America (MGFA), AChR, acetylcholine receptor, PSL, prednisolone.

^a^ Values were presented as medians (interquartile range).

^b^ *p* values were calculated using Fisher’s exact test.

^c^ *p* values were calculated using Mann–Whitney U tests.

Differences in sex and age were observed between the PSL and CNIs groups (*p* = 0.028 and *p* = 0.018, respectively). Baseline QMG scores, MG-ADL scores, MGFA classification, and AChR antibody titers were comparable between the two groups.

Individual clinical information is provided in Supplementary Table 1. One patient in the PSL group developed a thymoma and underwent thymectomy during hospitalization.

In the PSL group, PSL was administered on alternate days at a dose equivalent to 20–30 mg/day and tapered to 6–10 mg/day after one year. Three patients in the PSL group and one patient in the CNIs group received FT, including plasmapheresis or intravenous immunoglobulin (IVIg). CNIs were administered at doses of 1–3 mg/day for tacrolimus or 3 mg/kg/day for cyclosporine. Therapeutic drug monitoring was performed on all patients.

### 3.2 Improvement in MG-ADL and QMG scores at discharge

**Figure 2:**
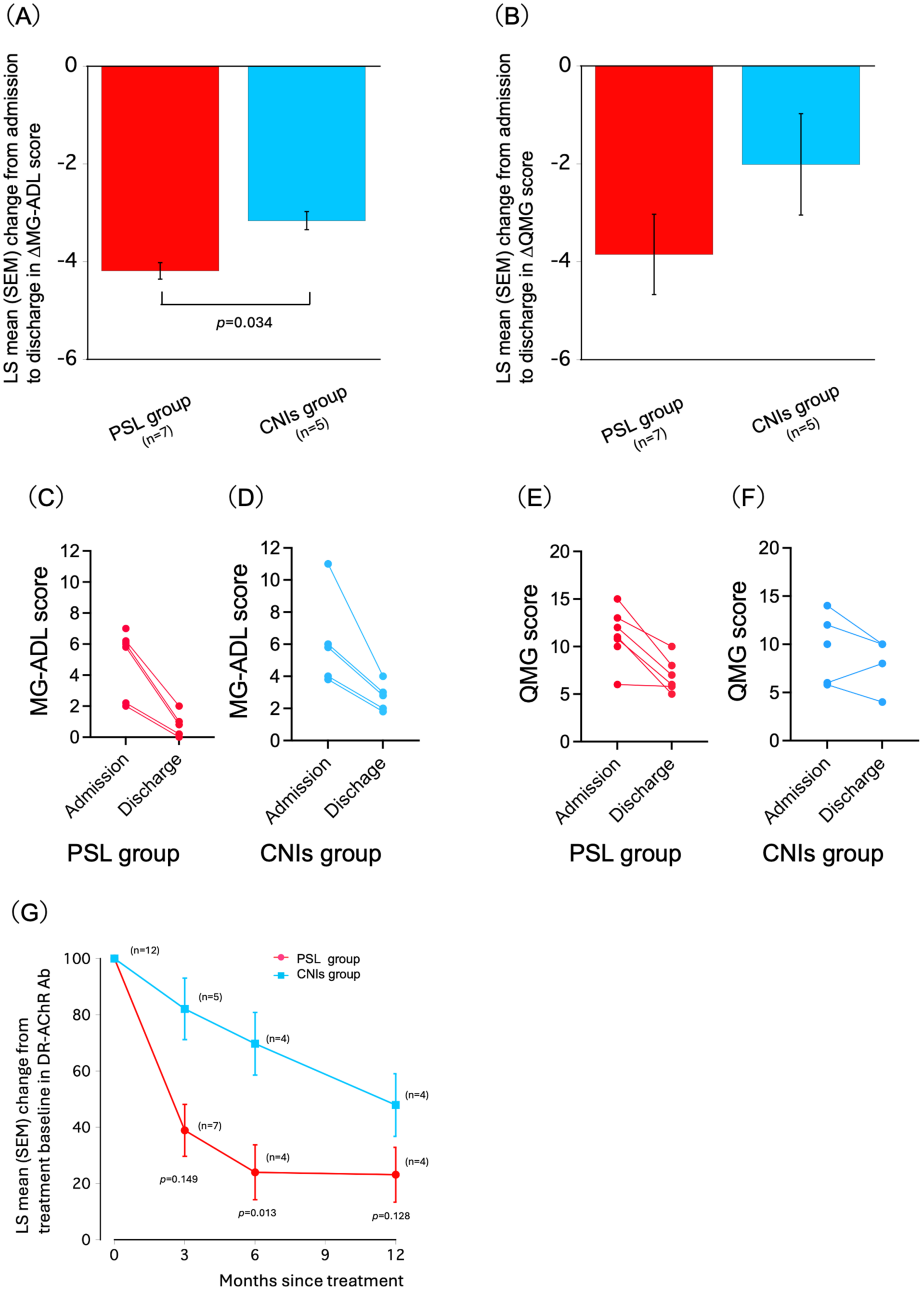
Least-squares (LS) mean changes (±SEM) in ΔMG-ADL, ΔQMG scores, and changes in MG-ADL and QMG scores at admission and discharge for each patient with MG and DR-AChR Ab levels at 3, 6, and 12 months between treatment groups. **(A)** ΔMG-ADL scores and **(B)** ΔQMG scores for each group, analyzed using a generalized linear model (GLM) with *a priori* selected covariates (fast-acting treatment [FT] and QMG score or MG-ADL score at admission). Missing data were imputed using multiple imputation by chained equations. *p*-values were obtained from a restricted maximum likelihood-based GLM, assessing differences in LS means between the two groups for ΔQMG and ΔMG-ADL scores. The ΔQMG score was calculated as the difference between QMG scores at admission and discharge; likewise, the ΔMG-ADL score was the difference between MG-ADL scores at admission and discharge. The graph shows changes in **(C,D)** the MG-ADL and **(E,F)** QMG scores at admission and discharge for each patient with MG in both groups. For cases with unavailable discharge data, only the admission values are shown **(G)** Baseline was defined as the initiation of oral immunosuppressant therapy. Changes from baseline were analyzed using a mixed model for repeated measures (MMRM), including group, month, and group × month interaction as fixed effects, with pre-treatment AChR-Ab titer as a covariate and subject as a random effect. Estimates were derived from the MMRM model. *p*-values were obtained using restricted maximum likelihood estimation to test for differences in LS means between treatment groups at each time point. CNIs, calcineurin inhibitors; DR-AChR Ab, decrement rate of acetylcholine receptor antibody titer from baseline; LS, least squares; MG, myasthenia gravis; MG-ADL, myasthenia gravis activities of daily living scale; PSL, prednisolone; QMG, quantitative myasthenia gravis; SEM, standard error of the mean.

The MG-ADL scores improved from admission to discharge in both groups (*p* = 0.005 for the PSL group; *p* = 0.024 for the CNIs group). Differences in the magnitude of change in the MG-ADL scores between the two groups were observed (p = 0.024). LS mean changes in ΔMG-ADL scores also differed between the groups (*p* = 0.034; Figure 2A).

QMG scores improved from admission to discharge in the PSL group (*p* = 0.016), whereas no statistically significant change was observed in the CNIs group (*p* = 0.352). Discharge QMG scores and LS mean changes in ΔQMG scores did not differ significantly between the groups (*p* = 0.449 and *p* = 0.233, respectively; Figure 2B). Sensitivity analyses yielded similar results (Supplementary Figure 1). Graphs showing the improvement in the MG-ADL and QMG scores in the groups treated with PSL and CNIs are shown in Figures 2C–F.

### 3.3 Decremental rate of AChR antibody titer (DR-AChR ab)

The MMRM analysis showed no overall difference in DR-AChR Ab levels between the groups over time (*p* = 0.129). A difference was observed at 6 months (*p* = 0.013), whereas no differences were detected at 3 or 12 months (*p* = 0.149 and *p* = 0.128, respectively; Figure 2G).

Sensitivity analysis using ANCOVA revealed group differences at 3 and 6 months (*p* = 0.024 and *p* = 0.016, respectively), which were not observed at 12 months (*p* = 0.058; data not shown).

### 3.4 Kaplan–Meier analysis for MM achievement

**Figure 3:**
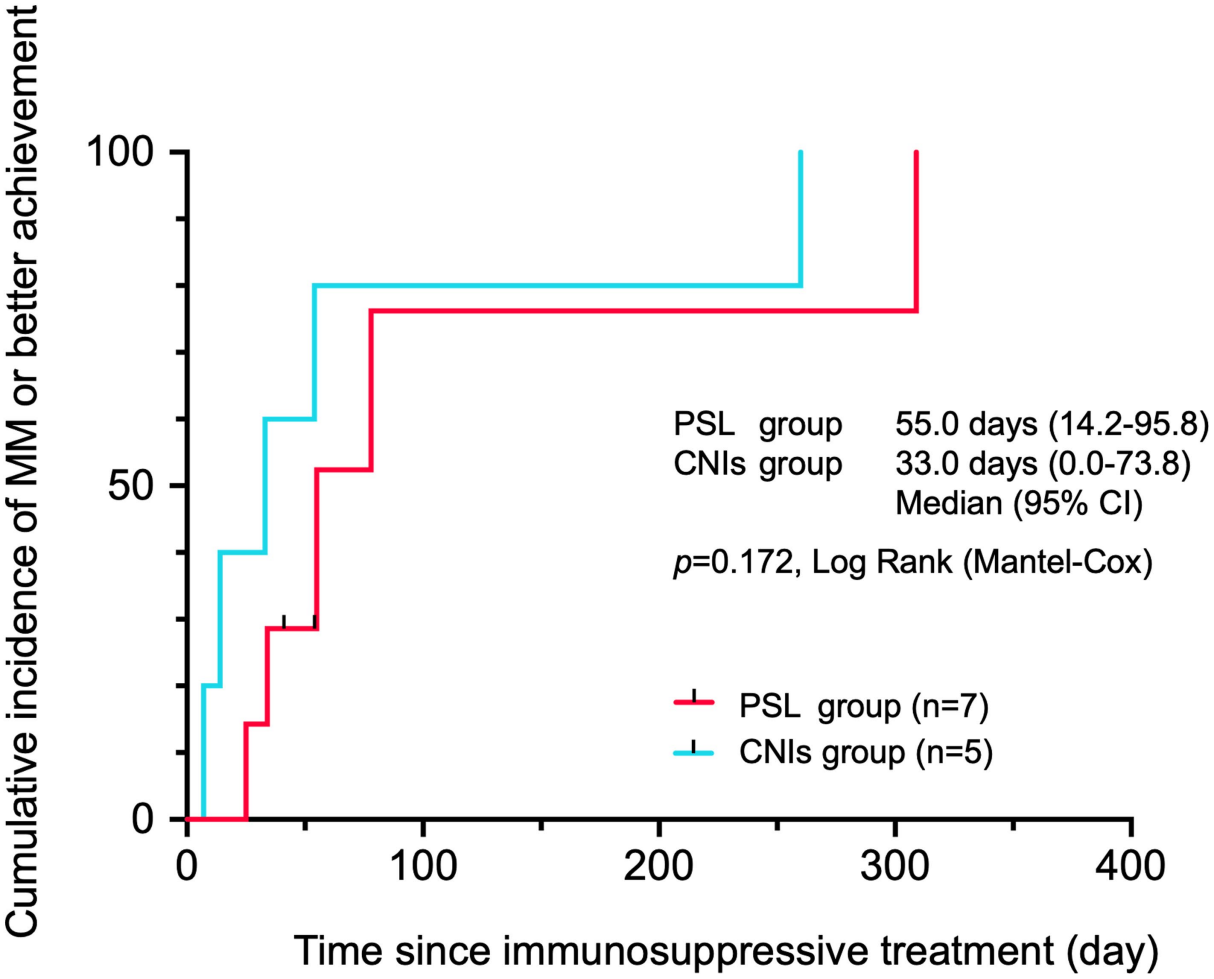
Cumulative incidence of minimal manifestation (MM) achievement in the PSL and CNIs groups. No significant difference in MM achievement was observed within 1 year of immunosuppressive treatment (*p* = 0.172, log-rank test). The two cases of loss to follow-up were due to hospital transfers. At the time of transfer, the patients were in a minimal symptom expression state (with MG-ADL score of 0 or 1). CNIs, calcineurin inhibitors; MM, minimal manifestation; PSL, prednisolone.

Kaplan–Meier curves for the time to MM or better status are shown in Figure 3. Although the log-rank test did not show a statistically significant difference between the groups (*p* = 0.172), the small sample size limits the interpretation of these findings. All patients in both groups achieved MM or better status within one year of treatment initiation. The median time to MM achievement was 55.0 days [95% confidence interval (CI): 14.2–95.8] in the PSL group and 33.0 days (95% CI: 0.0–73.8) in the CNIs group.

Detailed individual-level information on the timing and type of fast-acting treatment, length of treatment from immunosuppression initiation to discharge, and MG-ADL and QMG scores at admission and discharge are provided in Supplementary Table 1 to facilitate the transparent interpretation of clinical changes.

## 4 Discussion

In this retrospective cohort study, 18 of 46 hospitalized patients (39%) with acetylcholine receptor antibody (AChR-Ab)-positive MG were aged ≥ 70 years, which is consistent with the increasing prevalence of late-onset MG reported in previous epidemiological studies (2, 10, 11, 20). A recent epidemiological study reported that 26.8% of MG cases occur in patients aged ≥ 70 years (2). Older patients with MG are known to have a higher risk of treatment-related adverse events (7, 21), making optimization of immunotherapeutic strategies particularly important in this population.

Glucocorticoids remain the cornerstone of immunosuppressive therapy for MG (22, 23); however, they are associated with adverse effects in up to 66.7% of patients receiving PSL (14). These adverse effects, particularly in older adults, have been shown to negatively affect the quality of life in patients with MG (9, 14, 15). Regular PSL use is associated with a markedly increased risk of fractures and may adversely affect long-term outcomes (17, 18). Consequently, there is ongoing clinical interest in glucocorticoid-sparing approaches, including the use of nonsteroidal immunosuppressive agents (9, 15, 16, 23).

In this study, all patients aged ≥ 70 years achieved minimal manifestation (MM) or better status within one year, regardless of PSL use. No significant difference was observed in the cumulative incidence of MM achievement between the PSL and CNIs groups. Older patients with MG, particularly those aged ≥ 70 years, tend to have a more favorable prognosis and may respond well to immunosuppressive therapy (8, 11, 13, 24, 25). These findings suggest that disease control may be achieved without mandatory long-term prednisolone use in older patients with MG. Because patients in the CNIs group showed improvement in such a short period, it is important to consider not only the effects of CNI administration but also the effects of symptomatic treatment with pyridostigmine or the possibility that the condition followed its natural course. Interestingly, tacrolimus has also been reported to improve MG symptoms quickly by acting on ryanodine receptors to enhance excitation–contraction coupling (26).

However, given the small sample size and observational design, this interpretation should be considered an inductive inference derived from descriptive results rather than a confirmatory inference (6, 9, 10).

Patients in the PSL group demonstrated greater improvements in the MG-ADL and QMG scores at discharge, as well as transiently greater declines in AChR-Ab titers up to 6 months. These observations are consistent with the known immunological effects of PSL and suggest a role for PSL in achieving early symptom control. The absence of apparent differences in long-term clinical outcomes underscores the need to balance early efficacy with the risk of adverse events in older patients.

Disease exacerbation and respiratory deterioration may be less predictable in older patients with MG, increasing the risk of myasthenic crisis (4, 11). Although some patients in the present cohort achieved favorable outcomes without PSL, it is unlikely that all older patients will have a uniformly benign clinical course. From this perspective, therapeutic approaches that enable early symptom control with an acceptable safety profile may be clinically meaningful, particularly in the current era in which biological agents such as neonatal Fc receptor (FcRn) inhibitors are available (27, 28). Consideration of whether PSL is truly required in older patients while ensuring adequate early disease control may help inform future treatment strategies aimed at reducing crisis risk and treatment-related burdens (29). The study cohort spanned 2008 to 2018, a period before the widespread availability of modern biologics; therefore, the extent to which the findings are applicable to current clinical practice remains unclear. Although the results of this study do not allow us to draw conclusions about the efficacy of biological therapy in older patients with MG, future research on steroid therapy for this population may reveal that biological therapy could be an alternative to steroids for treating older patients with MG.

Although immunotherapy remains critical for preventing poor outcomes in older patients with MG (4, 30), CNIs have demonstrated efficacy and safety in this age group (9–13). These agents may serve as suitable long-term maintenance options, especially in patients intolerant to PSL or those transitioning from FcRn inhibitor therapy (31). However, it has been reported that overdose mainly increases the risk of side effects such as nephrotoxicity, neurotoxicity, infections, malignant tumors, diabetes, and gastrointestinal disorders; therefore, caution is needed when administering this medication to the elderly (27, 32).

Despite its strengths, this study has certain limitations. First, this was a single-center, retrospective analysis with a small sample size and no randomization. The high loss to follow-up and the lack of response-based outcomes may have reduced the robustness of the assessment. These results should therefore be interpreted as tentative hypotheses.

Second, there was the baseline imbalance between the treatment groups in terms of age and sex distribution. These differences likely reflect unmeasured clinical factors at treatment initiation, including comorbidities and physician preferences, which were not fully captured in this retrospective analysis. Residual confounding cannot be excluded, which limits causal inferences regarding treatment effectiveness. This study contained numerous biases in patient backgrounds and group comparisons, and could not recommend any particular treatment method for older adult patients.

Third, all patients achieved minimal manifestations within one year, indicating a drug-sensitive population. Therefore, our findings may not apply to patients with treatment-refractory MG. However, treatment response cannot be predicted at the disease onset. Our results suggest that some older patients with MG can achieve favorable outcomes without long-term PSL exposure, supporting a PSL-free initial treatment strategy in carefully monitored patients.

Fourth, this historical cohort study (2008–2018) preceded the availability of contemporary biological therapies, limiting the generalizability of the findings to current clinical practice.

Large-scale prospective studies incorporating contemporary treatments with longer follow-up periods are needed to validate PSL-free treatment strategies in older patients with MG.

Fifth, a major challenge in defining MM lies in its subjectivity. This is because the relevant guidelines do not specify which testing methods or criteria should be used to measure MM. Therefore, it was necessary to use minimal symptom expression (MSE), defined as a state in which the MG-ADL score is 0 or 1, as a measure of clinical symptoms.

## 5 Conclusion

In this small retrospective cohort of patients aged ≥70 years with myasthenia gravis, all patients achieved minimal manifestations within one year, regardless of prednisolone use. Given the limited sample size and observational nature of this study, these findings should be interpreted as descriptive and hypothesis-generating rather than confirmatory. Nonetheless, the results suggest that prednisolone may not be universally required to achieve disease control in all older patients with MG, highlighting the need for individualized treatment strategies and further prospective studies for validation.

## Data Availability

The original contributions presented in the study are included in the article/supplementary material, further inquiries can be directed to the corresponding author/s.
