# Supplementary material for: The treatment with or without prednisolone in adults aged ≥ 70 years with myasthenia gravis: a retrospective cohort study
Source: Front Neurol. 2026 Jul 29;17:1853517. doi: 10.3389/fneur.2026.1853517 (PMC13461386; doi:10.3389/fneur.2026.1853517)
Supplement: Supplementary file 1 [file Table_1.docx]

***Supplementary Material***

**Supplementary Figure.1A**

Violin plots with MG-ADL score at admission and at discharge in both groups.

Statistical analysis of multiple comparisons was performed the False Discovery Rate (FDR) approach after one-way ANOVA.

The confidence line showed medians, and dot lines showed the 25th and 75th quantiles.

MG-ADL: myasthenia gravis activities of daily living; PSL: Prednisolone; CNIs: calcineurin inhibitors.

**Supplementary Figure. 1B.**

Violin plots with QMG score at admission and at discharge in both groups.

Statistical analysis of multiple comparisons was performed the False Discovery Rate (FDR) approach after one-way ANOVA.

The middle line showed medians, and the dot lines showed the confidence of 25th and 75th quantiles.

QMG: quantitative myasthenia gravis; PSL: Prednisolone; CNIs: calcineurin inhibitors.
