# Supplementary material for: The treatment with or without prednisolone in adults aged ≥ 70 years with myasthenia gravis: a retrospective cohort study
Source: Front Neurol. 2026 Jul 29;17:1853517. doi: 10.3389/fneur.2026.1853517 (PMC13461386; doi:10.3389/fneur.2026.1853517)
Supplement: Supplementary file 2 [file Table_2.docx]

***Supplementary Material***

**Supplementary Table** . Detailed Information of Patients with Myasthenia Gravis over 70 years

|  | No | age | sex | MGFA | MG-ADL  (admission / discharge) | QMG  (admission / discharge) | Thymoma | Time from baseline to discharge (days) | AChR Ab （nM） | PSL maximum dosage (mg/AD) | CNIs | FT  (number of procedures) | Timing of FT (days from admission) | PB at discharge | Reasons for PSL avoidance | Underlying and Pre-existing conditions | Safety outcomes duration the observed period | Medications for underlying conditions | | | | | |
| --- | --- | --- | --- | --- | --- | --- | --- | --- | --- | --- | --- | --- | --- | --- | --- | --- | --- | --- | --- | --- | --- | --- | --- |
|  |  |  |  |  |  |  |  |  |  |  |  |  |  |  |  |  |  | PPI/H2 receptor antagonist | For osteoporosis | For HT | For HL | For DM | Other Medications |
| PSL+CNI group | 1 | 70 | M | IIa | 2 / 0 | 6 / 6 |  | 21 | 10.1 | 30 |  |  |  | PB |  | Hypothyroidism Left kidney donation | nothing to report | Esomeprazole | Alendronate Sodium Hydrate |  |  |  | Sulfamethoxazole/Trimethoprim |
|  | 2 | 70 | M | IIa | 7 / nd | 13/ 10 |  | 19 | 0.9 | 30 | TA  (1mg) | IAPP (4) | 21 – 27 |  |  | HT Multiple cerebral infarction past HBV infection | nothing to report | Esomeprazole | Alendronate Sodium Hydrate Menatetrenone | Amlodipine |  |  | Sulfamethoxazole/Trimethoprim Cilostazol |
|  | 3 | 73 | F | IIa | 2 / 0 | 15 / 8 |  | 54 | 18.7 | 20 | TA  (2mg) |  |  |  |  | HT HL | nothing to report | Rabeprazole | Alendronate Sodium Hydrate | Telmisartan |  |  | Difenidol |
|  | 4 | 73 | M | IIIb | 6 / 1 | 11 / 5 | B2 | 62 | 43.6 | 30 | TA  (3mg) | DFPP (3) IVIg (5) | 20 – 22  23 – 27 |  |  | HT Prostatic cancer  past HBV infection MCI | nothing to report | Esomeprazole | Eldecalcitol | Nifedipine |  |  | Sulfamethoxazole/Trimethoprim Bicalutamide Imidafenacin Olanzapine Entecavir |
|  | 5 | 75 | F | IIa | 6 / nd | 10 / nd |  | 58 | 24.4 | 25 | TA  (3mg) |  |  | PB |  | Regurgitation esophagitis Osteoporosis HL | nothing to report | Rabeprazole | Alendronate Sodium Hydrate |  | Simvastatin |  |  |
|  | 6 | 78 | M | IIIb | 5 / 1 | 12 / 7 |  | 20 | 39 | 30 | TA  (3mg) | PE (5) | 7 –21 |  |  | RS3PE DM HL Cerebral infarction Sleep apnea | nothing to report | Esomeprazole | Alfacalcidol、Menatetrenone | Nifedipine | Ravastatin | Metformin | Sulfamethoxazole/Trimethoprim Cilostazol |
|  | 7 | 79 | M | IIb | 6 / 2 | 11 / 6 |  | 42 | 27.3 | 30 | CY  (3mg/kg) |  |  | PB |  | DM CKD HT HL hyperuricemia Glaucoma BPH | nothing to report | Rabeprazole | Alendronate Sodium Hydrate | Nicardipine Irbesartan |  | Linagliptin | Sulfamethoxazole/Trimethoprim Bethanechol  Tamsulosin  Benzbromarone |
| CNI group | 8 | 74 | F | IIa | 6 / 3 | 12 / 10 |  | 7 | 25.3 |  | TA  (3mg) |  |  |  | underlying conditions | DM HL severe obesity Fatty liver Osteoporosis OA | nothing to report |  | Alendronate Sodium Hydrate |  | Simvastatin | Sitagliptin, Glimepiride, Pioglitazone, Voglibose | Ebastine Proheparum Rebamipide |
|  | 9 | 79 | F | IIb | 11 / 4 | 14 / 10 |  | 18 | 52.4 |  | TA  (2mg) | IAPP (4) | 13 – 22 | PB | underlying conditions | Osteoporosis Fraction of femoral neck Glaucoma IGT HL DVT | nothing to report | Rabeprazole | Alfacalcidol |  | Atorvastatin |  | Beraprost |
|  | 10 | 82 | F | I | 4 / 2 | 6 / 4 |  | 23 | 10.8 |  | TA  (2mg) |  |  | PB | aging, low back pain | Cerebral aneurysm MCI Chronic bronchitis Lumber spondylosis | nothing to report |  |  |  | Atorvastatin |  | Pregabalin Montelukast |
|  | 11 | 84 | F | IIa | 4 / 2 | 10 / nd |  | 14 | 25.2 |  | TA  (2mg) |  |  |  | underlying conditions | Osteoporosis Compression fractures of L1 and L4 MCI Fatty liver past infection of tuberculosis | nothing to report | Famotidine | Sodium Risedronate Hydrate Alfacalcidol |  |  |  | Aspirin Acetaminophen |
|  | 12 | 85 | F | IIa | 6 / 3 | 6 / 8 |  | 16 | 16.4 |  | TA  (2mg) |  |  | PB | aging | HT past HBV infection Osteoporosis Lumber spondylosis | nothing to report |  |  | Amlodipine Valsartan |  |  | Montelukast  Mirabegron |

PSL, prednisolone; CNI, calcineurin inhibitor; M, male; F, female; MGFA, myasthenia Gravis of Foundation of America; MG-ADL, myasthenia gravis activities of daily living ; QMG, quantitative myasthenia gravis ; AChR Ab; acetylcholine receptor antibodies; CY, Cyclosporine; TA, Tacrolimus; FT, early fast-acting treatments ; IVIg, intravenous immunoglobulin; IAPP, immunoadsorption plasmapheresis; DFPP, double filtration plasmapheresis; PE, plasma exchange; PB, pyridostigmine bromide ; HT, hypertension; HL, hyperlipidemia; HBV, hepatis B virus; MCI, mild cognitive impairment. DM, diabetes mellitus; RS3PE, remitting seronegative symmetrical synovitis with pitting edema; CKD, chronic kidney disease; BPH, benign prostatic hyperplasia; OA, osteoarthritis; IGT, impaired glucose tolerance; DVT, deep vein thrombosis; PPI, proton pump inhibitor; nd, no data.
